# Microstructural Evolution and Mechanical Properties of Cu–Ag Alloy via Different Severe Plastic Deformation Processes

**DOI:** 10.3390/ma18030581

**Published:** 2025-01-27

**Authors:** Haifeng Li, Haofeng Xie, Yizhi Zhao, Wenjing Zhang, Lue Huang, Yi Yuan, Hao Chu, Xujun Mi

**Affiliations:** 1 State Key Laboratory of Nonferrous Metals and Processes, GRINM Group Co., Ltd., Beijing 100088, China; xfcylhf@163.com (H.L.); xiehaofeng@grinm.com (H.X.); 18975151855@163.com (Y.Y.); mse.frozen@gmail.com (H.C.); 2 GRIMAT Engineering Institute Co., Ltd., Beijing 101407, China; 3 General Research Institute for Nonferrous Metals, Beijing 100088, China; 4 School of Materials Science and Engineering, South China University of Technology, Guangzhou 510640, China; yizhizhao1994@scut.edu.cn (Y.Z.); huangluehl@163.com (L.H.)

**Keywords:** Cu-Ag alloy, plastic deformation mode, microstructure, mechanical property, strengthening mechanism

## Abstract

The field of artificial intelligence and integrated circuits is experiencing rapid development, particularly in the area of highly integrated and miniaturized components, in which Cu-Ag alloys, as a typical lead frame material, play a crucial role. However, current research is primarily focused on low Ag content alloys, and there are few studies on the regulation of the microstructure and mechanical properties of high Ag content Cu-Ag alloys. This limitation hindered the development and utilization of the high Ag content Cu-Ag alloys. In this study, the microstructure and mechanical properties of Cu-28Ag (wt. %) alloy after room temperature and cryogenic rolling were investigated. It was demonstrated that the cryogenic rolling yielded better surface quality, an enhanced dendrite refinement effect, and a more distinct layer structure compared to room temperature rolling. The conductivity of the alloy decreased after cryogenic rolling due to increased electron scattering within the Cu matrix. The tensile strength improved, but the elongation decreased. Specifically, at a deformation amount of 95%, the alloy exhibited an ultimate tensile strength, yield strength, and elongation of 640 MPa, 631 MPa, and 1.9%, respectively. This strengthening was mainly attributed to the refinement of grains, the presence of dislocations, and precipitation. Furthermore, the samples subjected to liquid nitrogen rolling at a deformation amount of 95% exhibited improved homogeneous deformation capacity, which was attributed to grain size refinement, uniform distribution of high-density dislocations, deformation structure, and heterogeneity-induced deformation enhancement.

## 1. Introduction

The demand for durable and conductive copper wires and plates has increased due to the growing integration and miniaturization of components in the fields of artificial intelligence and integrated circuits [1,2,3]. In the Cu-Ag binary alloy system, the solubility of Ag in Cu is extremely low (less than 0.1 wt. % at room temperature). Typically, Ag precipitates prevent excessive scattering of electrons in the Cu matrix and provide a strengthening effect, which ensure the excellent electrical conductivity and high strength of the Cu-Ag alloys [4]. Cu-Ag alloys exhibit superior melting and casting processes compared to other alloy systems, such as Cu-Fe, Cu-Nb, and Cu-Cr [5,6,7], as well as broader adaptability to subsequent cold working. As a result, the Cu-Ag alloy system is widely used in electrical and microelectronic connectors, integrated circuit lead frames, and resistance welding electrodes [8].

The microstructure and properties of Cu-Ag alloys are influenced by factors like the Ag content, heat treatment techniques, and deformation processing methods. The Cu-Ag alloy is a typical eutectic system, and the maximum solubility of Ag in Cu is about 7.8 wt. % at 780 °C. Thus, when the added content of Ag is larger than 7.8 wt. %, the Cu-Ag alloy can be considered a high Ag content alloy, or else it is a low Ag content alloy [9]. The phase transformation of Cu-Ag alloys with different Ag content has been investigated during aging treatment. In low Ag content alloys, continuous precipitation occurs within the grains, resulting in the formation of nano-scale Ag-rich particles in the Cu matrix [10]. Additionally, there is a discontinuous precipitation reaction at the grain boundaries in high Ag content alloys, leading to the formation of a lamellar eutectic structure. Previous studies identified the interface of this reaction as a vulnerable point during stretching, which initiated cracks [11]. Therefore, current studies focus on Cu-Ag alloys with low Ag content, aiming to promote continuous precipitation reactions while suppressing the discontinuous precipitation reactions at grain boundaries. The purpose is to achieve the precipitation of high-density nano spherical Ag phases within the copper matrix, thereby enhancing the tensile strength of the alloy [12]. For example, Guo et al. found that adding a small amount of Cr to a Cu-6 wt. % Ag alloy suppressed the discontinuous precipitation reaction at grain boundaries, while promoting the precipitation of nano Ag particles within the grain, which led to improvements in the mechanical and electrical properties [13]. The deformation processing methods are also a crucial factor for the enhancement of mechanical properties of Cu-Ag alloy due to the high dislocation density and grain refinement. Xie et al. indicate that dislocation strengthening and boundary strengthening were the dominant strengthening mechanisms in the cold-rolled Cu–Ag alloys, and, meanwhile, the Ag addition increased the limiting concentration of dislocations and sub-grain boundaries in the Cu solid solution, resulting in higher steady-state strength in the cold-rolled Cu-Ag alloys than in pure copper [4].

In recent years, Cu-Ag alloys with high Ag content have demonstrated promising applications in key components of strong magnetic field and electronic information fields, including water-cooled magnets in pulsed/steady-state strong magnetic field devices and interconnected frameworks for large-scale integrated circuits. There are limited studies on the microstructure and mechanical properties of Cu-Ag alloys with high Ag content due to lamellar discontinuous precipitation, which hinders development and application.

In a recent study, Zhu et al. applied a “Bioinspired, heredity-derived hierarchical“ approach to systematically design Cu-Cr-Zr alloys with a hierarchical fibrous lamellar (HFL) structure [14]. This unique HFL structure primarily benefits from severe plastic deformation and avoids the potential drawbacks of dendrites by employing heat treatment techniques to ensure uniform composition and microstructure during traditional processing. In the HFL structure, the as-cast dendritic microstructure serves as the fundamental unit of multi-scale layered structures, greatly benefiting the mechanical properties of the alloy.

In Cu-Ag alloys with high Ag content, a significant amount of lamellar Ag-rich structures exist in the as-cast state. Transforming these structures into fine fibrous or lamellar structures through intense plastic deformation and utilizing them to form multilevel architectures can greatly enhance the alloy’s mechanical properties.

To summarize, the selection of an appropriate plastic deformation process is vital in controlling the layer structure of Cu-Ag alloys with high Ag content. This study extensively examined the impact of room temperature rolling and cryogenic rolling on the microstructure and mechanical properties of Cu-28Ag (wt. %) alloy, providing insights into the advantages and disadvantages of these two processes. Furthermore, the primary strengthening mechanism was discussed. The findings of this study will serve as a theoretical and experimental basis for the advancement of high-performance Cu-Ag alloys.

## 2. Experimental

In this work, the as-cast Cu-28Ag (wt. %) alloy was fabricated by German Vacuum Continuous Casting Machines OF Indutherm GmbH to produce the Cu-Ag alloy rod billet. The diameter of the copper alloy rod was 9 ± 0.2 mm. High-purity cathode electrolytic copper (99.995%) and high-purity Ag (99.99%) were melted and used as raw materials for the preparation of the mentioned Cu-Ag alloy. Atmosphere protection and water cooling were implemented during the downdrawing process. The as-cast samples then underwent a homogenizing treatment in a Muffle furnace at 680 °C for 2 h. Subsequently, room temperature rolling and cryogenic rolling were conducted, with deformation percentages of 85%, 90%, and 95% calculated as (thickness reduction/thickness of sample before rolling) × 100%.

The microstructure analysis of the alloy was conducted using a Bruker D8 ADVANCE X-ray diffractometer (XRD, D8 ADVANCE, Billerica, MA, USA) with a Cu target and Kα radiation (λ = 0.15406 nm). The scanning speed was set at 4 °/min, covering a range of 20~100°. Additionally, a FEI Titan G2 transmission electron microscope (TEM, FEI Titan G2, Waltham, MA, USA) was used for further microstructural observation. Scanning electron microscopy (SEM, FEI Quanta FEG 450, Waltham, MA, USA) and metallographic light microscopy (OM, Zeiss, Axiolab A1, Oberkochen, Germany) were employed to observe the microstructure. Metallographic samples were prepared by grinding with SiC sandpaper and subsequent polishing. The alloy surface was etched using an etching solution containing 3 g of FeCl_3_, 12.5 mL of HCl, and 56 mL of C_2_H_5_OH. TEM samples were thinned using TenuPlo-5 double spray thinner (Struers, Copenhagen, Denmark) with a voltage of 20 V and a temperature of −30 °C. Thin foils of the alloys for TEM were prepared via electropolishing in a twin-jet apparatus with a solution of 25% HNO_3_ + 75% CH_3_OH. Electron Back-Scattered Diffraction (EBSD) (Oxford Instrument, Symmetry S2, Oxford, UK) was performed to investigate the deformation microstructure, and the data were post-processed through the Channel 5 software. The EBSD samples were ion polished using a LEICA EM RES102 (Leica EM RES102, Leica Microsystems, Wetzlar, Germany) automatic ion thinning instrument. Electrical conductivity measurements were conducted using an eddy current conductivity meter (Sigma 2008B, Xiamen, China). Tensile properties of the alloy at room temperature were tested using an AGS-X 300kN micro-controlled electronic universal testing machine (AGS-X 300kN, Shimadzu, Kyoto, Japan) with a strain rate of 1 × 10^−4^ s^−1^. The original sample was cut into a tensile specimen size of (7 ± 0.3) mm × (1.5 ± 0.3) mm × (42 ± 0.3) mm (width × thickness × length) and polished with SiC sandpaper prior to testing.

## 3. Results

### 3.1. Microstructural Characterization

**Figure 1:**
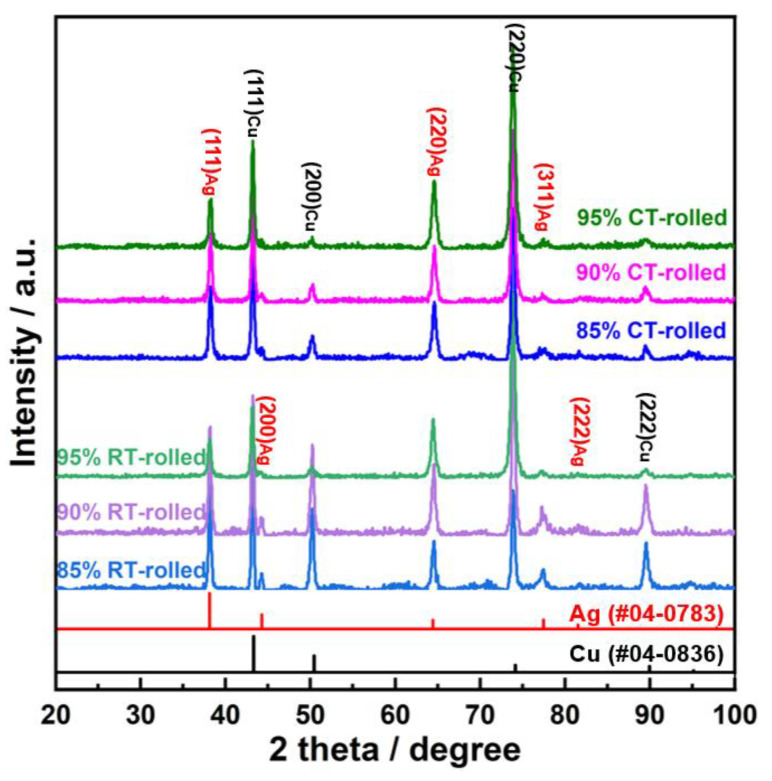
XRD (X-ray diffractometer) patterns of the Cu-28Ag (wt. %) alloy subjected to room temperature rolling and cryogenic rolling.

**Figure 2:**
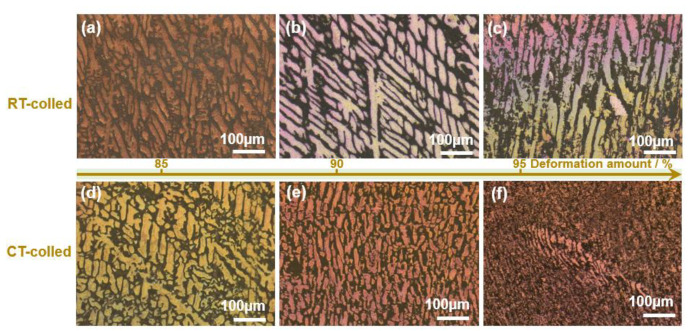
Metallographic structure of the Cu-28Ag (wt. %) alloy subjected to room temperature rolling (**a**–**c**) and cryogenic rolling (**d**–**f**).

**Figure 3:**
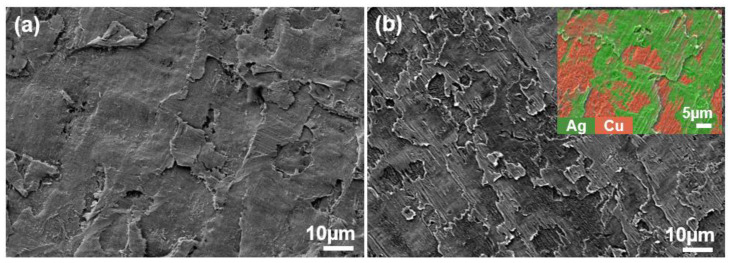
SEM (Scanning electron microscopy) secondary electron image of the Cu-28Ag (wt. %) alloy subjected to room temperature rolling (**a**) and cryogenic rolling (**b**) with a deformation of 95%.

The XRD (X-ray diffractometer) spectra of the alloys rolled at room temperature and in liquid nitrogen displayed distinct diffraction peaks for Cu and Ag, as illustrated in Figure 1. At room temperature, the solubility of Ag in Cu is very low; therefore, Cu-Ag alloys contain both Cu and Ag phases in the XRD spectra. Additionally, it is important to note that in the XRD patterns of all deformed samples, the intensity of the Cu (220) diffraction peak is the strongest, indicating a clear preferred orientation. Compared to the standard positions, the diffraction peaks of these alloys were shifted, indicating significant lattice distortion in Cu and Ag due to deformation. Figure 2 presents the metallographic structures of the alloys rolled at room temperature and in liquid nitrogen, revealing a typical dendritic structure. The gray–white areas represent dendrite cores, while the black areas indicate dendrite boundaries, attributed to the higher corrosion resistance of the cores. With increased deformation, the dendritic structure became elongated and significantly refined. Compared to room temperature rolling, the elongation and refinement of dendrites were more pronounced in the cryogenic rolled alloys. To further investigate the microstructural differences between alloys rolled at room temperature and in liquid nitrogen, SEM (Scanning electron microscopy) analysis was conducted on alloys with 95% deformation, as shown in Figure 3. Compared with the room-temperature-rolled alloy, the deformation bands in the cryogenic rolled alloys were evidently superior and dense, exhibiting more severe deformation. This was attributed to the effective suppression of the dynamic recovery of face-centered cubic metals at the extremely low temperature of liquid nitrogen, greatly enhancing the severe plastic deformation of the metal [15]. Additionally, EDS mapping analysis revealed an alternating distribution of Cu and Ag, which increased the Cu/Ag interface density, thereby enhancing the inhibition of dislocation motion.

**Figure 4:**
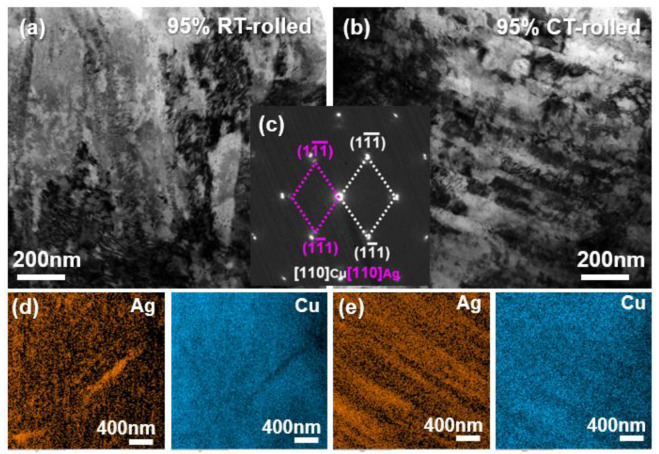
Bright field (BF) image (**a**,**b**), the corresponding selected area electron diffraction (SAED) pattern (**c**), and the corresponding Energy Disperse Spectroscopy (EDS) mapping (**d**,**e**) of the Cu-28Ag (wt. %) alloy subjected to room temperature rolling and cryogenic rolling with a deformation of 95%.

Figure 4 presents the TEM (transmission electron microscope) analysis results of alloys rolled at room temperature and in liquid nitrogen with a deformation of 95%. Both alloys exhibited a high density of dislocations, with proliferating dislocations entangling with each other, indicating that the alloy primarily underwent dislocation slip during severe deformation. The selected area electron diffraction (SAED) results revealed that the alloy’s microstructure contained only Cu and Ag phases, with a clear orientation relationship: [110]_Ag_//[110]_Cu_, (1$\overset{-}{1}$1)_Ag_//(1$\overset{-}{1}$1)_Cu_. Additionally, some diffraction spots appeared as arcs, indicating that the fibrous crystals exhibited a strong {110} texture and a pronounced ⟨111⟩ orientation. Combined with the EDS (Energy Disperse Spectroscopy) mapping analysis results, it was concluded that both the room temperature and cryogenic rolled alloys exhibited a Cu/Ag layered structure, with the latter being more pronounced and uniform.

### 3.2. Mechanical Properties

**Figure 5:**
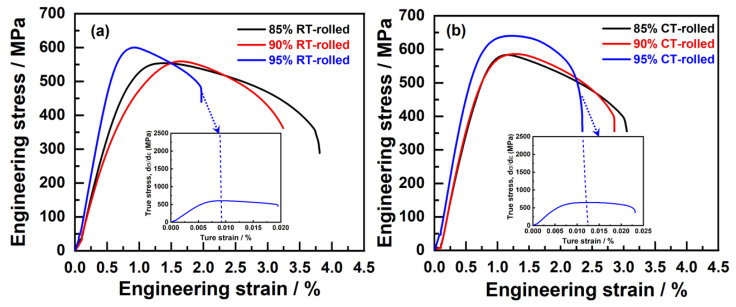
Tensile engineering stress–strain curves of the alloys subjected to room temperature (RT) rolling (**a**) and cryogenic (CT) rolling (**b**) (the inset is the corresponding strain-hardening rate (SHR) curves of the alloys after rolling with a deformation of 95%).

Figure 5 illustrates the room temperature engineering stress–strain curves of Cu-28Ag (wt. %) alloys rolled at both at room temperature and 77 K (in liquid nitrogen), with deformation amounts of 85%, 90%, and 95%. As the deformation amount increases, the ultimate tensile strength (*σ*_UTS_) and yield strength (*σ*_YS_) of the room-temperature-rolled alloy rose from 553 ± 15 MPa to 602 ± 18 MPa and from 506 ± 12 MPa to 597 ± 18 MPa, respectively, while the elongation (*δ*) decreased from 3.15 ± 1.21% to 1.45 ± 1.02% (Table 1). The primary reason for the increase in alloy strength was the generation of numerous dislocations, which inevitably affected the deformation capability. Furthermore, the stress–strain curves of these alloys exhibited an almost non-existent yield plateau, indicating insufficient uniform deformation capability. Compared with the room-temperature-rolled alloys, the cryogenic-rolled alloys exhibited similar trends in *σ*_UTS_, *σ*_YS_, and *δ* %. With increasing deformation, the *σ*_UTS_ and *σ*_YS_ of the cryogenic-rolled alloys rose from 583 ± 10 MPa to 640 ± 20 MPa and from 539 ± 21 MPa to 631 ± 25 MPa, respectively (Table 1), showing a significantly higher strength increase compared to the room-temperature-rolled alloys. This indicated that cryogenic rolling was able to enhance the work hardening capacity of the alloy effectively, suppress the dynamic recovery of dislocations, and thus produce alloys with a high density of dislocations, providing favorable conditions for subsequent annealing heat treatment. When the deformation amount was 85% and 90%, the elongation of the cryogenic-rolled alloys was lower than that of the room-temperature-rolled alloys, and there was still no yield plateau exhibited in the stress–strain curves. However, when the deformation amount reached 95%, the *δ* of the cryogenic-rolled alloys exceeded that of the room-temperature-rolled alloys, and a yield plateau appeared, indicating that the alloys exhibited some uniform deformation capability. The strain-hardening rate (*θ*) was the rate of change on the true stress as a function of true strain during plastic deformation [16], also known as the strain change modulus, which more clearly reflected uniform elongation, as shown in the insets of Figure 1. The strain-hardening rates of cryogenic- and room-temperature-rolled alloys dropped sharply, but the uniform elongation of the former was higher than that of the latter. In addition, the changes in conductivity of the alloys are shown in Table 1, with the conductivity of the cryogenic-rolled alloys being lower than that of the room-temperature-rolled alloys. When the deformation amount increases from 85% to 90%, the conductivity slightly decreases, while it significantly decreases when the deformation amount reaches 95%. This suggests that low-temperature rolling leads to a stronger deformation effect and a higher dislocation density, resulting in an increased number of electron scattering centers, which reduces the conductivity of the alloy, and the deformation effect is most pronounced at 95% deformation.

## 4. Discussion

Based on the analysis of microstructure and mechanical properties mentioned above, the tensile strength of alloys rolled in cryogenic and at room temperature significantly increased, but this inevitably decreased the elongation. During the tensile testing of room-temperature-rolled alloys, no uniform deformation stage was observed. However, when the deformation reached 95% through cryogenic rolling, the alloy had high tensile strength and exhibited a certain degree of uniform deformation, providing favorable conditions for further structural control. Therefore, the deformation structures of cryogenic rolling and room temperature rolling alloys with a deformation amount of 95% will be compared to further elucidate their strengthening mechanisms.

### 4.1. Strengthening Mechanism of the Cryogenic Rolling Process

**Figure 6:**
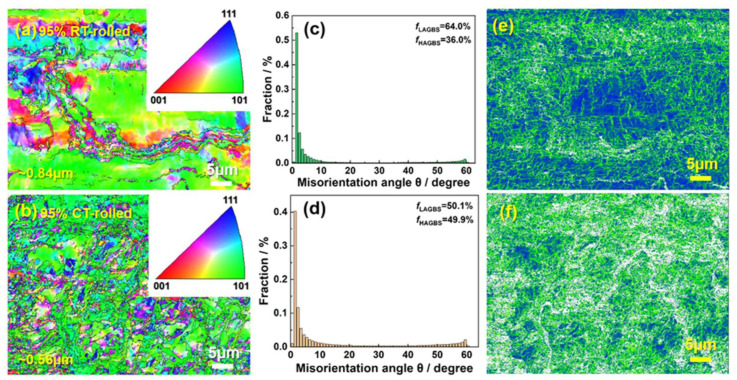
Inverse pole figure (**a**,**b**), the corresponding grain boundary type distribution (**c**,**d**), and the corresponding kernel average misorientation map (**e**,**f**) of the Cu-28Ag alloy subjected to room temperature rolling and cryogenic rolling with a deformation of 95%.

Figure 6 shows the EBSD (Electron Back-Scattered Diffraction) analysis results of cryogenic-rolled and room-temperature-rolled alloys with a deformation of 95%. The grains of the room-temperature-rolled alloy are elongated and unevenly distributed, with a size of ~0.84 ± 0.05 µm. The analysis of grain boundary types shows that the proportion of high-angle grain boundaries in the alloy is 36.0%, while the proportion of low-angle grain boundaries is 64.0%. Compared to room-temperature-rolled alloys, the grain refinement of cryogenic-rolled alloys is more obvious and the distribution is more uniform, at ~0.56 µm, and the proportion of low-angle grain boundaries in the alloy is reduced. Reducing the grain size can provide a fine-grain strengthening effect, which is beneficial for improving tensile strength. High grain boundary density can enhance the ability to coordinate deformation, thereby ensuring alloy plasticity [17]. In addition, the larger the grain size, the more significantly increased the winding activity during the post-uniform deformation process, leading to premature failure and lower uniform elongation. Therefore, the alloys rolled at room temperature have almost no uniform elongation. It is worth noting that low-angle grain boundaries have lower energy and higher grain boundary strength [18]. However, although the proportion of low-angle grain boundaries in the alloys rolled at room temperature is higher, their elongation is still lower than that of the cryogenic-rolled alloy, indicating that not only do we need to pay attention to the proportion of different grain boundary types but we also need to further clarify the grain boundary density. The kernel average misorientation (KAM) maps of the two alloys are shown in Figure 6e,f. The average orientation difference is related to the geometrically necessary dislocation density, and the higher the KAM value, the higher the dislocation density [19]. The KAM value of the cryogenic-rolled alloys and the room-temperature-rolled alloy are 0.34° and 0.28°, respectively. Obviously, the dislocation density and distribution of cryogenic-rolled alloys are higher and more uniform, providing a more significant dislocation strengthening effect. Meanwhile, a uniform distribution of dislocation density reduces the possibility of stress concentration.

**Figure 7:**
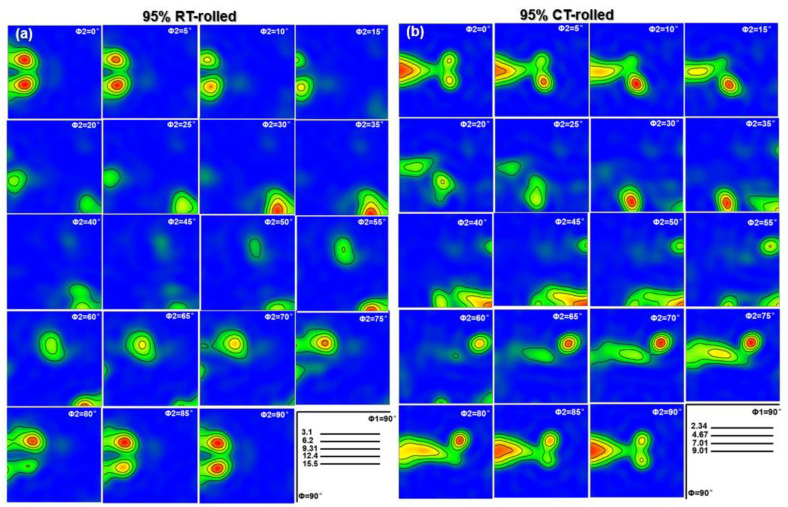
Orientation distribution function (ODF) of the Cu-28Ag (wt. %) alloy subjected to room temperature rolling (**a**) and cryogenic rolling (**b**) with a deformation of 95%.

The texture represents the preferred orientation of the deformed structure, and it also has a significant impact on the mechanical properties of the alloy. When alloy grains are stacked and arranged along a specific orientation, high-density grain boundaries are formed, thereby increasing the tensile strength of the material [20]. Furthermore, this specific grain arrangement can also give the material higher plasticity and better resistance to plastic deformation. The TEM analysis results have confirmed the existence of a deformation texture in the Cu-28Ag alloy. Further EBSD results more finely display the texture of room temperature rolling and cryogenic rolling alloys, and the evolution of the macrotexture is illustrated by constant ϕ2 sections of the Orientation distribution function (ODF), as shown in Figure 7. It is important to note that the evolution of the ODF image is closely related to the crystal structure. Given that Cu has an FCC crystal structure, the orientations exhibit high symmetry when the ϕ2 equals 0 and 90°, resulting in similar ODF images. For ϕ2 = 0°, the main texture type is {110}<001>, and the strength is the highest. For ϕ2 = 45°, {110}<001> still exists, but the strength is weak. For ϕ2 = 65°, a strong {123}<634> texture appears. Similarly, the cryogenic rolled alloy also exhibits a typical rolling structure with ϕ2 = 0°, forming a {110}<001> (Goss) texture with high strength, while there is also a weaker {110}<112> (Brass) structure. For ϕ2 = 45°, the {110}<112> texture still exists, while the {110}<001> texture disappears, forming the {112}<111> (Copper) texture. For ϕ2 = 65°, a strong {123}<634> (S) texture is formed, that is, the texture distribution of the Cu-28Ag alloy after cryogenic rolling with a deformation of 95% mainly includes {110}<001> (Goss), {110}<112>, and {112}<111> textures. Based on the above analysis, the texture of the cryogenic-rolled alloy is significantly stronger than that of the room-temperature-rolled alloy. The strong selection of the Cu-28Ag alloy provides stronger dislocation resistance during the tensile process, thereby improving the alloy’s strength and work-hardening ability.

In addition to grain refinement, uniform high-density dislocation distribution, and selective selection, the deformed Cu-Ag alloy has a layered structure with significant differences in hardness between Cu and Ag, forming an alternating state of soft and hard regions. For this layered heterogeneous structure, the strain during the tensile process is uneven but continuous. The Cu/Ag interface generates a strain gradient, forming high-density geometric dislocations, which induce deformation-induced long-range internal stress, namely hetero-deformation-induced (HDI) stress. This provides a sustainable self-hardening mechanism for the Cu-28Ag alloy and ensures its uniform deformation ability [21].

### 4.2. Multiple Strengthening Effect Contributions to the Yield Strength

In the present study, the Cu-28Ag (wt. %) alloy exhibited a significant amount of Ag precipitation and underwent severe plastic deformation, indicating the simultaneous introduction of fine-grain strengthening, dislocation strengthening, and precipitation strengthening mechanisms in the alloy, which was consistent with the EBSD analysis results. Furthermore, it is worth noting that the solubility of Ag in Cu is extremely low under room temperature conditions. As a result, the solid solution strengthening effect is neglected. Here, taking the cryogenic rolled alloy with a deformation of 95% as an example, the three strengthening effects are analyzed to clarify the contributions of different strengthening mechanisms. It needs to be noted that the calculation parameters used in the strength calculation are those of pure copper due to the low solubility of Ag in the Cu-Ag alloy.

(1) Fine-grain strengthening

The grain size is fine, and the number of grain boundaries is increased. Grain boundaries produce resistance to dislocation motion, the effect of which is calculated using the Hall–Patch formula, as follows [22,23,24]:

(1) $$\sigma_{\mathrm{gb}}=k_{\left(r\right)}d^{-\frac{1}{2}}$$

where the strengthening coefficient, denoted as *k*_(r)_, is equal to 0.18 MPa·m^1/2^ [25]. The grain size of the cryogenic-rolled alloy with a deformation of 95% is ~0.56 µm, which means that the strength increment brought by the fine-grain strengthening effect of the alloy is ~240 MPa.

(2) Dislocation strengthening

The strength increment caused by dislocation strengthening (*σ_dis_*) is estimated using the following equation [26,27]:

(2) $$\;\sigma_{dis}=M\alpha Gb{\left(\rho_{d}\right)}^{-1/2}$$

where the Taylor factor, denoted as *M*, is equal to 3.06 for pure metals or alloys with a face-centered cubic structure. Additionally, α represents a geometric constant of approximately 0.3 for the Cu alloy [28], *G* is the shear modulus of the copper matrix and equal to 46 GPa [29], and *b* is Burgers vector (0.2556 nm for the Cu alloy [28]). The dislocation density is denoted as *ρ*_d_. Based on the analysis results of the KAM image (orientation difference of 0.34°), the estimated value is ~4.0 × 10^−15^/m^2^. Based on this calculation, the strength increment brought by this part is ~170 MPa.

(3) Precipitation strengthening

The Ag phase is distributed in a layered form in the cryogenic-rolled alloy with a deformation of 95%; thus, the strengthening effect is closely related to interlayer spacing, *d_w_*, expressed by the following equation [30,31]:

*σ*_p_ = *k*(2*d_w_*)^−1/2^ (3)

where the *d_w_* is the average distance between the lamellae and *k* is a constant assumed to be 0.14 for the copper alloy [31]. The *d_w_* of the cryogenic-rolled alloy with a deformation of 95% is ~240 nm; thus, the contribution of discontinuous precipitation strengthening is ~200 MPa, respectively. It should be noted that as the strength calculations are based on empirical formulas and the parameters involved in the calculations cannot be accurately obtained, there may be discrepancies between the calculated values and the actual data. The strength calculation results show that the contributions of fine-grain strengthening, dislocation strengthening, and precipitation strengthening in the alloy after cryogenic rolling are basically equivalent, with the contribution of fine-grain strengthening slightly higher.

## 5. Conclusions

In the present study, the influence of room temperature rolling and cryogenic rolling on the microstructure, electric conductivity, and mechanical properties of the Cu-28Ag alloy with high Ag content was systematically studied. The sources of mechanical properties and strengthening mechanisms of the alloy were further discussed. The specific conclusions are as follows:

(1) After room temperature and cryogenic rolling, the dendritic crystals of the Cu-28Ag alloy are significantly refined, and the Ag phase is distributed in fibrous layers. Cryogenic rolling improves the surface quality and dendritic refinement effect of the alloy, and the lamellar structure is more pronounced, indicating that this method is an effective means of regulating the microstructure of Cu-Ag alloys with high Ag content in the as cast state.
(2) The conductivity of the alloy decreases after room temperature rolling and cryogenic rolling, mainly due to the increase in electron scattering particles in the matrix. The tensile strength of the alloy increases, but its elongation decreases. When the deformation of cryogenic rolling is 95%, the alloy exhibits good comprehensive mechanical properties, with ultimate tensile strength, yield strength, and elongation of 640 MPa, 631 MPa, and 1.9%, respectively.
(3) The strengthening mechanism of the Cu-Ag alloy includes fine-grain strengthening, dislocation strengthening, and precipitation strengthening, and the results of the strength calculations show that the strength increments due to different strengthening mechanisms are basically equivalent, with the contribution of fine-grain strengthening being slightly higher.
(4) After cryogenic rolling with a deformation amount of 95%, the alloy has a certain degree of uniform deformation ability, which is attributed to grain refinement, high-density dislocations with uniform distribution, the deformation texture, and the improvement in the heterogeneous induced deformation ability.

## Figures and Tables

**Table 1:** The ultimate tensile strength, yield strength, and elongation of the alloys subjected to room temperature rolling and cryogenic rolling.

Alloy	σ_UTS_/MPa	σ_YS_/MPa	δ/%	Electric Conductivity/%IACS
85% RT-rolled	553 ± 15	506 ± 12	3.15 ± 1.21	86.45 ± 1.15
90% RT-rolled	561 ± 13	473 ± 20	2.60 ± 0.90	85.38 ± 0.75
95% RT-rolled	602 ± 18	597 ± 18	1.45 ± 1.02	82.40 ± 0.85
85% CT-rolled	583 ± 10	539 ± 21	2.55 ± 1.32	79.55 ± 0.95
90% CT-rolled	588 ± 16	542 ± 22	2.35 ± 0.95	79.04 ± 1.28
95% CT-rolled	640 ± 20	631 ± 25	1.90 ± 1.50	69.63 ± 0.85

## Data Availability

The data presented in this study are available on request from the corresponding author.
